# Optimizing Traumatic Limb Salvage: Ectopic Implantation and Staged Rotationplasty

**DOI:** 10.3390/medicina59101879

**Published:** 2023-10-23

**Authors:** Yi-Keng Hsieh, Chang-Heng Liu, Ching-Hsuan Hu

**Affiliations:** 1 Department of Plastic and Reconstructive Surgery, Chang Gung Memorial Hospital, Chang Gung Medical College, Chang Gung University, Taoyuan City 333, Taiwan; liyaunking@gmail.com; 2 Department of Orthopaedic Surgery, Chang Gung Memorial Hospital, Linkou Medical Center, Taoyuan City 333, Taiwan; elepheng@gmail.com; 3 Bone and Joint Research Center, Chang Gung Memorial Hospital, Linkou Medical Center, Taoyuan City 333, Taiwan

**Keywords:** rotationplasty, ectopic implantation, limb salvage, trauma

## Abstract

Rotationplasty, a limb-saving procedure involving a 180-degree ankle rotation to function as a knee joint, is now standard for treating distal femur osteosarcoma. However, challenges related to self-identification persist within the Asian population. This study presents a case involving the successful application of temporary ectopic implantation followed by staged rotationplasty after a severe traumatic amputation, resulting in a favorable outcome. Additionally, a systematic review is conducted to summarize the various difficulties and complications encountered in different studies. This approach improves the feasibility of rotationplasty in traumatic cases and enhances patient and family comprehension.

## 1. Introduction

Rotationplasty is a limb-sparing surgical procedure that involves a 180-degree rotation of the ankle, effectively transforming it into a “new knee” [1]. This innovative technique gained recognition as the standard treatment for osteosarcoma of the distal femur after its pioneering execution by Salzer in 1981 [1,2]. Rotationplasty boasts several advantages, including enhanced gait patterns, increased walking speeds with reduced energy expenditure compared to total amputations, and the retention of the ability to use a below-knee prosthesis, which offers superior functionality over an above-knee prosthesis [3]. These advantages enable patients to embrace a better quality of life. Nowadays, this technique is starting to be applied to traumatic patients, with pleasing results [3,4,5,6]. However, it is important to note that self-acceptance of rotationplasty remains a challenge in the Asian population. Many individuals in Asian cultures are more conservative and may struggle to embrace the concept of a reversed ankle joint. Consequently, rotationplasty is rarely performed, even in cases of lower extremity malignant bone tumor reconstruction [5,7]. Furthermore, there are ongoing challenges documented in the literature that both patients and surgeons may encounter throughout the treatment process [3,4,5,6].

In a different context, the technique of temporary ectopic banking of an amputated limb is well-established. It is employed in situations where immediate replantation is unfeasible due to poor tissue condition, extensive injuries, or a high risk of complications. Ectopic implantation holds the potential to salvage and restore the amputated limb at a later point in time [8,9]. Additionally, ectopic implantation allows for adequate time for the surrounding tissues to settle and aids in infection control [8,9].

In this study, we present a case of limb salvage involving temporary ectopic implantation, followed by staged rotationplasty after a severe traumatic amputation, alongside a comprehensive review of the existing literature.

## 2. Case Report

**Figure 1:**
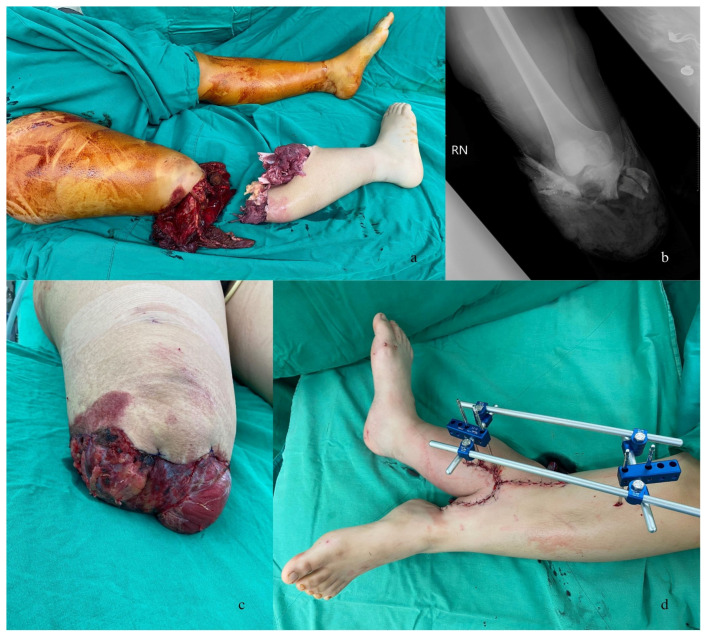
Right lower leg traumatic amputation after traffic accident. (**a**) Intraoperative photo demonstrates extensive soft tissue destruction. (**b**) The radiograph shows the tibial plateau articular-comminuted fracture. (**c**) Intraoperative photo shows the soleus and gastrocnemius muscles were utilized to cover the distal femur. (**d**) Immediately postoperative, the ectopic implantation procedure was completed.

A 19-year-old healthy female had a motorbike accident in which she slipped and suffered from a complete below-knee amputation (Figure 1a,b). She was immediately taken to the emergency department. Upon arrival, she had a Glasgow Coma Scale score of 15, and her initial examination revealed cardiovascular stability.

Due to the severe crush injury and contamination of the amputated stump, primary replantation was not possible. However, given the intact and uninjured ankle joint, traumatic rotationplasty was considered. At that point, her family expressed hesitations regarding rotationplasty, primarily due to aesthetic concerns. Given the family’s concerns, emergency surgery for ectopic replantation was performed to preserve the residual limb. In this procedure, the proximal amputated stump of the right lower limb was debrided, and residual fragments of the fibula and tibia were removed. The soleus and gastrocnemius muscles were then utilized to cover the distal femur (Figure 1c).

The amputated part underwent shortening of the tibia and fibula by 10 cm, along with the removal of all unhealthy muscle and foreign bodies. External skeletal fixation (ESF) was then applied to temporarily stabilize the right lower leg to the left leg. Subsequently, a thorough examination identified the tibial nerve, sural nerve, deep peroneal nerve, posterior tibial artery (PTA), anterior tibial artery (ATA), vena comitans, and greater saphenous vein (GSV) for further anastomosis. Following the identification of nerves and vessels, another surgical team explored the left lower leg for recipient vessels. In the end, the right posterior tibial artery was anastomosed to the left posterior tibial artery (PTA), and the accompanying vein of the posterior tibial artery was also anastomosed to the accompanying vein of the left PTA. Three superficial veins were also anastomosed (Figure 1d).

**Figure 2:**
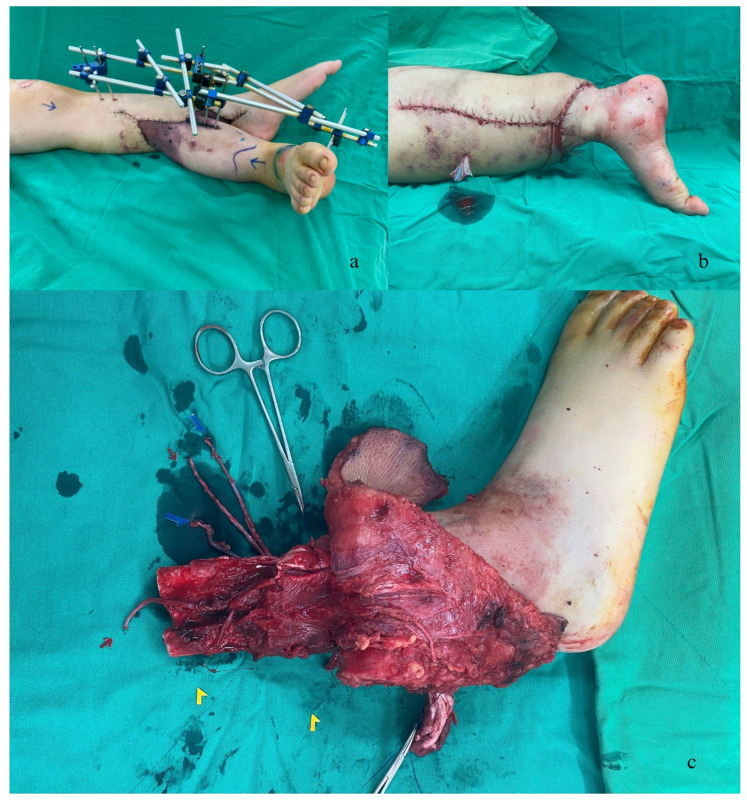
(**a**) The skin paddle of the implanted right lower leg exhibited signs of congestion. (**b**,**c**) The main structure was tagged and prepared for repair, and rotationplasty was completed. (yellow arrow: tibial nerve, deep peroneal nerve; red arrow: anterior tibial artery, posterior tibial artery; blue arrow: concomitant veins of posterior tibial artery).

After three days, a second-look operation was performed to resect additional necrotic tissue and to assess the condition of all vessels. We discovered that the previous superficial veins were all occluded, and only the concomitant veins remained patent. Therefore, we decided to excise the occluded superficial veins. The skin paddle of the amputated right lower leg showed signs of congestion, so we applied vacuum-assisted closure at 125 mmHg in intermittent mode (Figure 2a). A third look at the vessel condition was performed three days later in preparation for the rotationplasty. We opened the wound on the left leg and found that the posterior tibial artery (PTA) and concomitant vein were patent, but there was a significant amount of fibrinous tissue surrounding the PTA and concomitant vein, which made the vessels adhere to peripheral tissue, making dissection difficult. Consequently, we removed all fibrinous tissue and freed the vessels, after which the wound was primarily closed.

The scheduled rotationplasty was performed on the 10th day post-injury, following thorough discussions with the patient and her family. Initially, the external skeletal fixation (ESF) on the previously ectopically implanted leg was removed. The implanted leg was divided after vital structures, such as the posterior tibial artery (PTA), anterior tibial artery (ATA), two concomitant veins of the PTA, deep peroneal nerve, tibial nerve, and sural nerve, were identified. The PTA of the contralateral leg was repaired after the implanted leg was divided. The orthopedic surgeon then prepared the right thigh femur bone. The popliteal artery, popliteal vein, tibial nerve, common peroneal nerve, and saphenous nerve were also identified. The fixation of the tibia and femur bones was carried out by the orthopedic surgeon, with an overlap of about 8 cm and double plating at the superior and lateral sites. An autologous bone graft was harvested from the ipsilateral femoral condyle and inserted into the fixation area.

Subsequently, we proceeded with vascular and nerve reconstruction. The PTA was anastomosed to the popliteal artery, the ATA was anastomosed to a branch of the popliteal artery, the two concomitant veins of the PTA were anastomosed to the popliteal vein and its branch, the tibial nerve was anastomosed to the tibial nerve, the deep peroneal nerve was anastomosed to the common peroneal nerve, and the sural nerve was anastomosed to the saphenous nerve. Regarding the muscles, the patella was removed; the quadriceps were attached to the Achilles tendon; the vastus lateralis muscle was connected to the flexor hallucis longus (FHL); the biceps and semimembranosus muscles were connected to the tibialis anterior muscle; the semitendinosus was connected to the extensor hallucis longus (EHL); and the gracilis muscle was attached to the peroneus longus. An ICG test was performed, confirming that all vessels were patent, and all wounds were primarily closed (Figure 2b,c).

After the surgery, the patient was transferred to the intensive care unit (ICU) for further management. She was then transferred to the ward in stable condition ten days after the rotationplasty and was discharged 14 days after the procedure.

**Figure 3:**
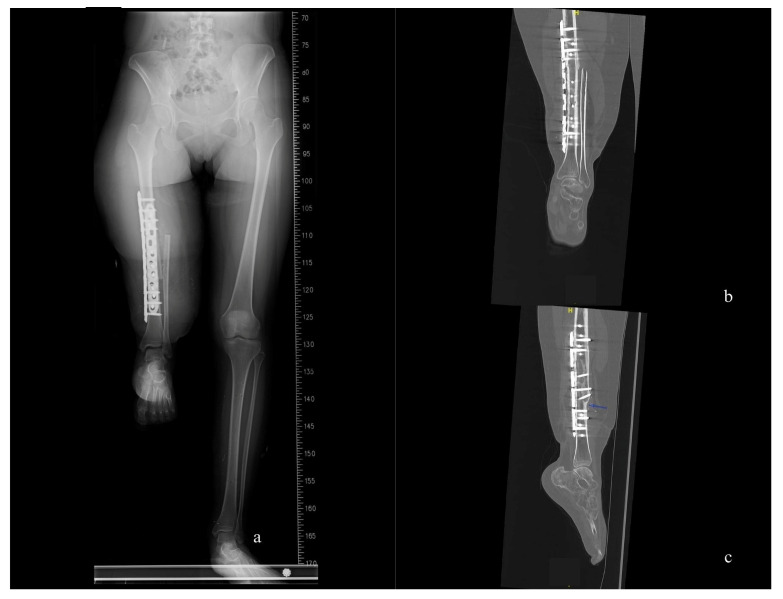
The position of the right “new knee” is appropriate. (**a**) Whole-leg axis radiographs at the two-months follow-up. (**b**,**c**) CT scans were conducted at the twelve-month follow-up, revealing complete healing of the bone union site and a straight alignment of the leg axis in coronal and sagittal view.

**Figure 4:**
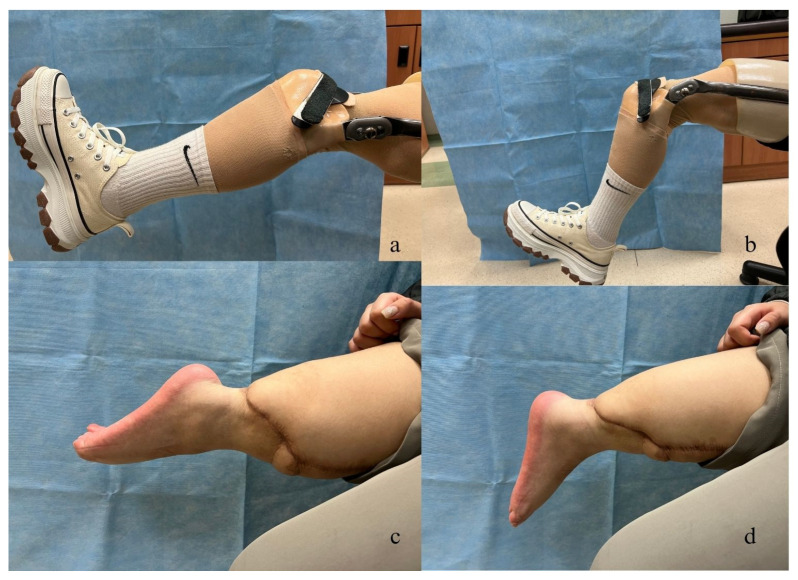
(**a**) Range of motion of the prosthetic knee joint: 20° of extensor lag. (**b**) Range of motion of the prosthetic knee joint: maximum flexion (70°). (**c**) Range of motion of the ankle joint: maximum plantar flexion (70°). (**d**) Range of motion of the ankle joint: maximum dorsiflexion (0°).

After two months, we conducted a follow-up X-ray of the surgical site, which revealed bone union and appropriate alignment of the leg axis. Five months after the rotationplasty, the patient demonstrated excellent ambulation and could independently climb stairs at the twelve-month follow-up (Figure 3 and Figure 4, and Supplementary Video S1, Figures S1 and S2). Regarding sensory recovery, the patient reported hypersensitivity and pain (visual analog score: 5) over the plantar area of the reversed knee at the two-month follow-up. However, sensation significantly improved without pain at the eighteen-month follow-up, with tactile sensation now present over the entire foot and ankle. The two-point discrimination test over the plantar and heel area measured 4 cm.

## 3. Discussion

In this study, we present a case that underwent ectopic implantation followed by staged rotationplasty. Ectopic implantation was first reported by Godina et al. [8] in 1986 when they successfully implanted a nonvascularized left hand in the right axilla temporarily and replanted it after nine weeks. Nowadays, with the advancements in microsurgery, ectopic implantation has become a valuable technique for salvaging amputated limbs [8,9,10].

Theoretically, immediate rotationplasty potentially carries a high risk of infection due to severe wound bed contamination, which disrupts tissue perfusion and wound healing and reduces bone union rates [5,11,12]. Due to the uncertainty regarding the severity of the bone injury at the time of the trauma, non-union of the bones after immediate traumatic rotationplasty is also quite common [5]. In our case, performing ectopic implantation provided enough time to optimize the recipient wound bed and bone condition [10,11,12]. Additionally, we were able to use an autologous bone graft harvested from the ipsilateral femoral condyle at the same time when the bone was relatively healthy. This may explain why the bone union time was shorter compared to the reported cases [13,14].There are several aspects in which utilizing ectopic implantation may prove beneficial. Not only does it afford us the time needed for tissues to settle down, but also during this period, the surgical team, psychological counselors, physiotherapists, and prosthetists can engage in discussions with the patient and their family, ensuring the patient’s motivation to undergo rotationplasty. Shared decision-making can occur concurrently, allowing patients and their families ample time to comprehend the clinical situation and make a well-informed decision. This not only grants the patient and their family the right to make a medical decision with a rational mindset but also enables them to be well-prepared for rotationplasty, which is an uncommon procedure with an unconventional postoperative appearance [5].

The quality of vessels before performing anastomosis for rotationplasty is also a crucial consideration [15]. In our case, during the ectopic replantation procedure, we discovered that several vessels were occluded during the second and third evaluations. Consequently, we conducted multiple excisions of the occluded vessels and performed several vessel anastomoses. We believe that conducting a second or third evaluation for assessing the quality of vessels is an important step during ectopic implantation, as inflammation of injured vessels can trigger thrombus formation [16].

During these subsequent evaluations, we had the opportunity to remove unreliable and occluded vessels, while the ones that remain patent can be used as donor vessels for rotationplasty later. Apart from evaluating vascular quality, tissue adherence is another critical factor that affects the harvesting of the implanted limb. Tissue adhesions refer to abnormal attachments that form between tissues and vessels [17]. While the exact mechanism of postoperative tissue adhesion is not fully understood, ischemia and inflammation have long been recognized as factors that can stimulate adhesion [18]. This aligns with our observations on postoperative day 7 when we noted a significant amount of fibrinous tissue surrounding the PTA and concomitant vein, causing these vessels to adhere to peripheral tissue. Therefore, we recommend that staged rotationplasty should be performed once the wound bed is stabilized, before the donor vessels adhere to peripheral tissue.

According to our literature review, there are only four reported cases of rotationplasty performed after severe trauma, each with its own set of difficulties and complications (refer to Table 1). Krettek et al. [6] presented a case involving a 31-year-old male who sustained a grade-IIIC open, AO type-33C3.3 fracture of the left femur’s distal part in a train accident. Three months after the injury, rotationplasty was performed on his left leg. At the eighteen-month follow-up, the patient could walk five kilometers without assistance and reported no pain. However, he faced challenges due to limited knee flexion, particularly when getting in and out of automobiles. Bone union was achieved in four months.

Klos et al. [4] reported the case of an 80-year-old male who suffered from severe circumferential degloving and avulsion of the entire soft tissue sleeve from the left hemipelvis to the knee after being struck by a truck. Ten days after the traumatic event, he underwent rotationplasty, but the results showed a limited range of motion and several complications, including tactile hypoesthesia in the left lower limb, weakness of the tibialis anterior muscle, and depression during rehabilitation. Bone union was noted at the two-year follow-up.

Tye et al. [3] presented a case involving a patient who was hit by an automobile while skateboarding on the road, resulting in a Gustilo–Anderson Type IIIA open right distal femoral shaft fracture. The patient underwent rotationplasty after the failure of intramedullary nail fixation due to infection. After rotationplasty, the patient was able to ambulate well with a prosthesis at the 13-month follow-up, and bone union was achieved by eight months.

Lu et al. [5] reported a case of a patient who suffered a left femur shaft fracture with a left tibial plateau articular comminuted fracture that extended into the diaphysis due to a gaseous explosion while cutting gas barrels. The patient underwent rotationplasty immediately after the injury, and the results were satisfying. The patient could ambulate independently with a prosthesis at the four-year follow-up. However, complications such as a lack of understanding of the rotationplasty procedure before surgery and a request for amputation immediately after surgery, bone nonunion that required further revision surgery at eighth months post-surgery, and left foot numbness that limited walking distance were noted. Bone union was achieved in 11 months.

To sum up, traumatic rotationplasty after lower-leg malignant tumor resection presents major concerns, including infection, bone nonunion, sensory impairment, limited range of motion, and the need for long-term rehabilitation when compared to conventional rotationplasty. There is still significant room for improvement in traumatic rotationplasty to achieve outcomes comparable to those of conventional rotationplasty.

## 4. Conclusions

In conclusion, we recommend initiating the process with ectopic implantation before proceeding with rotationplasty after severe traumatic events. This approach optimizes the condition of the wound bed and bone, ultimately reducing the risk of complications. Additionally, it allows ample time for the patient and their family to make a well-informed decision with a clear state of mind, benefiting both the patient and the surgical team.

## Figures and Tables

**Table 1:** Studies of severe traumatic injuries that underwent rotationplasty.

Reference	Gender, Age (yr.)	Injured Mechanism	Lower Limb Injured Type	Results	Bone Union Time	Complications
Krettek et al., 1997 [6]	M, 31	As a passenger in a train accident	Open fracture of the distal portion of the left femur	Can ambulate with prothesis	3 months	Limited range of motion while getting into and out of vehicles Unsatisfying walking pattern
Klos et al., 2010 [4]	M, 80	Knocked off by a truck while biking	Severe degloving wound from left hemipelvis to the knee	Limited range of motion	2 years	Left lower limb tactile hypoesthesia Weakness of the tibialis anterior muscle Depression during rehabilitation
Tye et al., 2021 [3]	M, 26	Struck by an automobile	Open right distal femoral shaft fracture	Can ambulate with prothesis	8 months	-
Lu et al., 2022 [7]	M, 37	Gaseous explosion	Left femur shaft fracture and tibial plateau articular comminuted fracture	Can ambulate with prothesis	11 months	Not clear of the procedure before surgery Second surgery for bone fixation Left foot numbness

M = male.

## Data Availability

The data are unavailable due to privacy or ethical restrictions.

## Supplementary Materials

The following supporting information can be downloaded at https://www.mdpi.com/article/10.3390/medicina59101879/s1, Video S1: The video captures the patient during a one-year follow-up. The patient exhibits a comfortable gait cycle with a slight circumduction gait. Figure S1 and Figure S2: Right lower leg x-ray at the 6-month follow-up.

## References

[1] Gupta S.K., Alassaf N., Harrop A.R., Kiefer G.N. (2012). Principles of rotationplasty. J. Am. Acad. Orthop. Surg..

[2] Salzer M., Knahr K., Kotz R., Kristen H. (1981). Treatment of osteosarcomata of the distal femur by rotation-plasty. Arch. Orthop. Trauma Surg..

[3] Tye E.Y., Taylor A.J., Combs K., Kay R.D., Bryman J.A., Hoshino C.M. (2021). Early Post-Traumatic Van Nes Rotationplasty After an Open Femur Fracture With a Necrotizing Soft-Tissue Infection. J. Bone Jt. Surg..

[4] Klos K., Mückley T., Gras F., Hofmann G.O., Schmidt R. (2010). Early Posttraumatic Rotationplasty After Severe Degloving and Soft Tissue Avulsion Injury: A Case Report. J. Orthop. Trauma.

[5] Lu C.-K., Liu Y.-C., Chen C.-T., Fu Y.-C., Liu W.-C. (2022). Immediate rotationplasty for a severely crushed floating knee in a blast injury: A case report. Trauma Case Rep..

[6] Krettek C., Lewis D.A., Miclau T., Schandelmaier P., Lobenhoffer P., Tscherne H. (1997). Rotationplasty for the Treatment of Severe Bone Loss and Infection of the Distal End of the Femur. A Case Report. J. Bone Jt. Surg..

[7] Wang H., Lu Y., Chien S., Lin G., Lu C. (2003). Rotationplasty for Limb Salvage in the Treatment of Malignant Tumors: A Report of Two Cases. Kaohsiung J. Med. Sci..

[8] Godina M., Bajec J., Baraga A. (1986). Salvage of the Mutilated Upper Extremity with Temporary Ectopic Implantation of the Undamaged Part. Plast. Reconstr. Surg..

[9] Cho B.H., Higgins J.P. (2019). Revascularization and Replantation in the Hand: Ectopic Banking and Replantation. Hand Clin..

[10] Yoshida S., Koshima I., Narushima M., Nagamatsu S., Yokota K., Yamashita S., Harima M. (2019). Usefulness of ectopic implantation in multiple finger amputation injury. Clin. Case Rep..

[11] Jain A.K., Sinha S. (2005). Infected Nonunion of the Long Bones. Clin. Orthop. Relat. Res..

[12] Sibbald R.G., Orsted H., Schultz G.S., Coutts P., Keast D. (2003). International Wound Bed Preparation Advisory Board, Canadian Chronic Wound Advisory Board, Preparing the wound bed 2003: Focus on infection and inflammation, Ostomy. Wound. Manag..

[13] Pape H.C., Evans A., Kobbe P. (2010). Autologous Bone Graft: Properties and Techniques. J. Orthop. Trauma.

[14] Miller C.P., Chiodo C.P. (2016). Autologous Bone Graft in Foot and Ankle Surgery. Foot Ankle Clin..

[15] Tan B.-K., Fong H.C., Tan E.-K., Raj J.P. (2021). Strategies for a successful hepatic artery anastomosis in liver transplantation: A review of 51 cases. Ann. Acad. Med. Singap..

[16] Davis C., Fischer J., Ley K., Sarembock I.J. (2003). The role of inflammation in vascular injury and repair. J. Thromb. Haemost..

[17] Dizerega G.S. (1994). Contemporary adhesion prevention. Fertil. Steril..

[18] Tsai S.-W., Fang J.-F., Yang C.-L., Chen J.-H., Su L.-T., Jan S.-H. (2005). Preparation and Evaluation of a Hyaluronate-Collagen Film for Preventing Post-Surgical Adhesion. J. Int. Med. Res..

