# Supplementary figures and images for: Optimizing Traumatic Limb Salvage: Ectopic Implantation and Staged Rotationplasty
Source: Medicina (Kaunas). 2023 Oct 23;59(10):1879. doi: 10.3390/medicina59101879 (PMC10608738; doi:10.3390/medicina59101879)

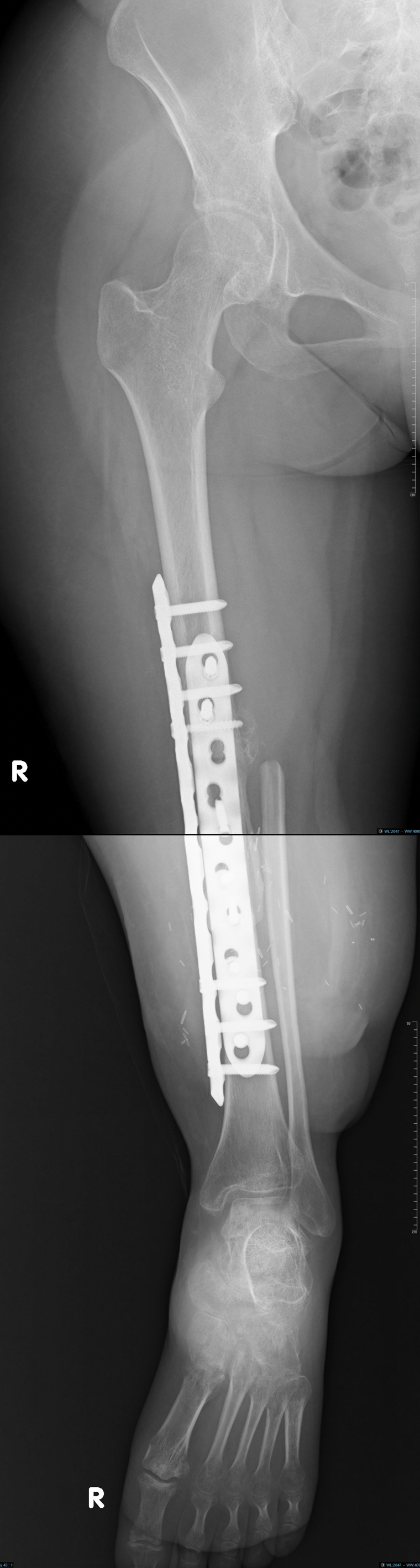

Supplement: Supplementary file 1 [file medicina-59-01879-s001.zip › supplement/Figure S2.tiff]

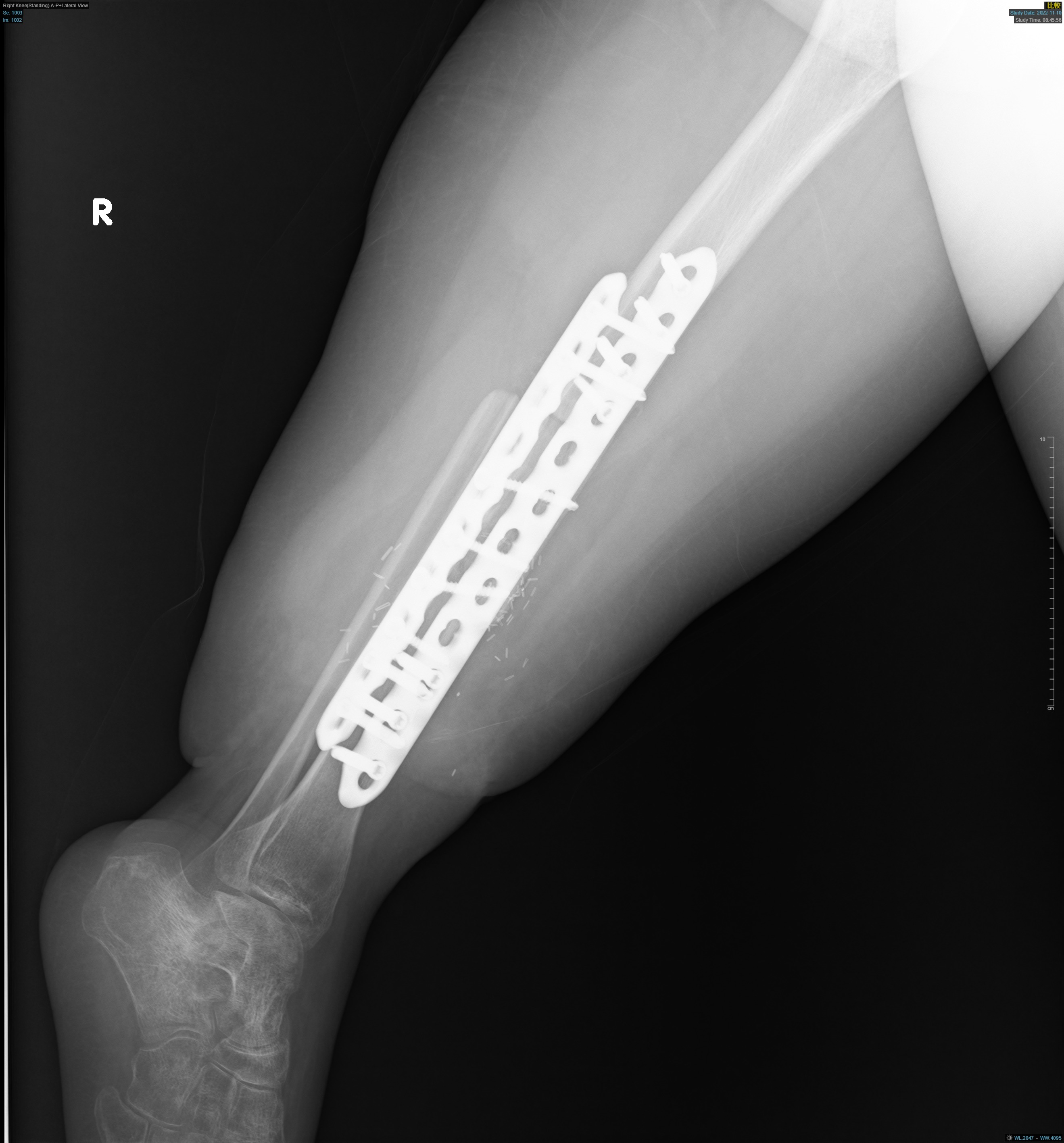

Supplement: Supplementary file 1 [file medicina-59-01879-s001.zip › supplement/Figure S1.JPG]
